# Phage Encoded H-NS: A Potential Achilles Heel in the Bacterial Defence System

**DOI:** 10.1371/journal.pone.0020095

**Published:** 2011-05-18

**Authors:** Connor T. Skennerton, Florent E. Angly, Mya Breitbart, Lauren Bragg, Shaomei He, Katherine D. McMahon, Philip Hugenholtz, Gene W. Tyson

**Affiliations:** 1 Advanced Water Management Centre, The University of Queensland, St. Lucia, Queensland, Australia; 2 Australian Centre for Ecogenomics, School of Chemistry and Molecular Biosciences and Institute for Molecular Bioscience, The University of Queensland, St. Lucia, Queensland, Australia; 3 College of Marine Science, University of South Florida, Saint Petersburg, Florida, United States of America; 4 CSIRO Mathematics, Informatics and Statistics, St. Lucia, Queensland, Australia; 5 Department of Energy Joint Genome Institute, Walnut Creek, California, United States of America; 6 Energy Biosciences Institute, University of California, Berkeley, California, United States of America; 7 Department of Civil and Environmental Engineering, University of Wisconsin at Madison, Madison, Wisconsin, United States of America; J. Craig Venter Institute, United States of America

## Abstract

The relationship between phage and their microbial hosts is difficult to elucidate in complex natural ecosystems. Engineered systems performing enhanced biological phosphorus removal (EBPR), offer stable, lower complexity communities for studying phage-host interactions. Here, metagenomic data from an EBPR reactor dominated by *Candidatus* Accumulibacter phosphatis (CAP), led to the recovery of three complete and six partial phage genomes. Heat-stable nucleoid structuring (H-NS) protein, a global transcriptional repressor in bacteria, was identified in one of the complete phage genomes (EPV1), and was most similar to a homolog in CAP. We infer that EPV1 is a CAP-specific phage and has the potential to repress up to 6% of host genes based on the presence of putative H-NS binding sites in the CAP genome. These genes include CRISPR associated proteins and a Type III restriction-modification system, which are key host defense mechanisms against phage infection. Further, EPV1 was the only member of the phage community found in an EBPR microbial metagenome collected seven months prior. We propose that EPV1 laterally acquired H-NS from CAP providing it with a means to reduce bacterial defenses, a selective advantage over other phage in the EBPR system. Phage encoded H-NS could constitute a previously unrecognized weapon in the phage-host arms race.

## Introduction

Phage, viruses that infect bacteria and archaea, play a fundamental role in the environment through predation and lateral gene transfer [1]. Uncultured environmental phage have been most extensively studied in marine ecosystems where they have been demonstrated to affect oceanic biogeochemistry [2]. The importance of phage has been recognized in other environments including many engineered systems that are often low diversity and susceptible to phage attack. For example in the dairy industry, phage induced collapse of the fermentation process cause significant economic loss [3]. Engineered systems also provide an ideal environment for investigation of phage-host dynamics in less complex communities under controlled conditions. One such system, wastewater treatment, relies on a process known as enhanced biological phosphorus removal (EBPR; [4]) to remove dissolved organic carbon and phosphorus. However, wastewater treatment plants performing EBPR can suffer from unpredictable loss of performance, which can lead to large discharges of phosphorus into waterways [5]. Recent culture-independent studies of EBPR have mostly focused on the biology of the dominant member of the community, *Candidatus* Accumulibacter phosphatis (CAP), including a complete genomic characterization [6]. Due to their dominance, CAP populations are susceptible to ‘kill-the-winner’ [7] predation by phage. However, despite the potential involvement of phage in the loss of EBPR performance [8], genomic characterization of EBPR phage populations to help understand phage-host interactions is lacking.

Microorganisms have developed a number of methods to defend against phage attack. Extracellular polysaccharides form a first layer of defense by providing a physical barrier against phage entry [9]. Phage can subvert this by using degradative enzymes to reach host cell receptors. Once the phage genome has been injected into the host cell, restriction modification systems can target and degrade phage DNA [10]. Phage have been shown to evade Type III restriction by corrupting recognition sequences in their genome [11]. CRISPRs (clustered regularly interspersed short palindromic repeats) are the most recently discovered phage defense mechanism and act as a type of adaptive immune system. Bacteria and archaea incorporate small fragments of phage genomes into their CRISPR loci as spacers between repeats, which are then used to direct degradative protein machinery against future infecting phage [12]. A CRISPR spacer must be identical to the phage genome sequence for resistance [13] and can therefore be a potent driving force for phage evolution.

Recent reports have revealed that CRISPR expression can be regulated by a histone-like nucleoid structuring (H-NS) protein in *Escherichia coli* [14], [15], [16]. H-NS is a global bacterial repressor protein primarily found in the Proteobacteria [37] and has been studied extensively due to its widespread effect on the transcriptome [17], [18]. Genome wide analysis has determined that H-NS preferentially binds to regions of high AT-content and it has been suggested that H-NS regulates the expression of variable elements in the genome such as transposases or horizontally acquired genes [19].

Here, we present the first analysis of a phage metagenome from an EPBR environment. One of the dominant phage genomes assembled from the metagenomic sequence data encodes H-NS, the first discovery of this repressor on a phage genome. The presence of H-NS suggests a previously unrecognized mechanism for evasion of host defense mechanisms, including CRISPRs.

## Results and Discussion

### Phage metagenome assembly and community composition

A conventionally operated enhanced biological phosphorus removal (EBPR) bioreactor was sampled by random shotgun sequencing on two occasions seven months apart [20]. At the first sampling time (t_0_), total microbial biomass (bacteria, archaea and phage) was sequenced and analyzed as previously reported [6], and at the second time point (t_7_) purified phage virions were sequenced. While the t_0_ microbial metagenome was extensively analyzed [6], the t_7_ phage metagenome was only screened for molecular links between the phage and host populations [20]. Here we assembled the t_7_ dataset (16 Mbp of Sanger sequence) using two assembly methods (Table S1) and a consensus was built using overlapping contigs from each assembly. Despite the small size of the t_7_ dataset, two thirds of the data assembled into only 13 contigs, comprising three complete and six partial phage genomes (Table 1; Figure S1) out of ∼130 genotypes estimated by PHACCS [21]. This indicates the presence of a small number of dominant EBPR phage types consistent with the microbial community structure which is dominated by a single uncultured bacterial population, *Candidatus* Accumulibacter phosphatis (CAP) [6].

**Table 1:** Characteristics of assembled EBPR phage phage metagenome from sampling time t_7_.

Phage ID	Total contig length (bp)	Estimated genome completeness^3^	% GC	Coverage± standard deviation^4^	Relative abundance^5^	Number of ORFs (percentage annotated)	Putatative host	Viral family	Accession	Temperate	Number of genotypes
EPV1^1^	36,119	Complete	59.5	11.7±4.1	8.5	55 (27)	CAP^6^	*Podoviridae*	JF412294	Yes	1(+1)^8^
EPV2	40,628	Complete	63.9	12.8±5.3	9.2	68 (25)	Unknown	*Podoviridae*	JF412295	Yes	1
EPV3	38,991^2^	Partial	41.8	7.3±3.7	5.3	53 (5)	Unknown	*Podoviridae*	JF412296	No	N.D.^9^
ESV1	54,511	Complete	55.1	10.0±3.9	7.4	82 (12)	Unknown	*Siphoviridae*	JF412297	No	2
ESV2	48,954	Partial	62.9	29.4±9.2	21	77 (24)	CAP^7^	*Siphoviridae*	JF412298	No	1
ESV3	13,588	Partial	61.7	30.9±10.0	22.1	13 (23)	Unknown	*Siphoviridae*	JF412299	No	1
ESV4	10,050	Partial	65.4	6.4±2.6	4.6	17 (41)	Unknown	*Siphoviridae*	JF412300	No	N.D.^9^
ESV5	13,119	Partial	63.0	21.4±8.1	15.6	26 (11)	Unknown	*Siphoviridae*	JF412301	No	1
ESV6	11,444	Partial	66.8	5.8±2.4	4.2	16 (6)	Unknown	*Siphoviridae*	JF412302	No	N.D.^9^

1 Present in t_0_ and t_7_.

2 Represented by four contigs.

3 Estimated completeness was based on an inventory of required structural genes present in the contig.

4 Figure given is the average read depth at each position ± standard deviation.

5 Relative abundance in the t_7_ phage metagenome is estimated based on GAAS output.

6 Host based on the presence of H-NS in the genome.

7 Host based on the presence of a CAP CRISPR spacer match in the genome.

8 One genotype found in t_7_ plus another genotype found in t_0_.

9 Not determined due to low coverage.

**Figure 1:**
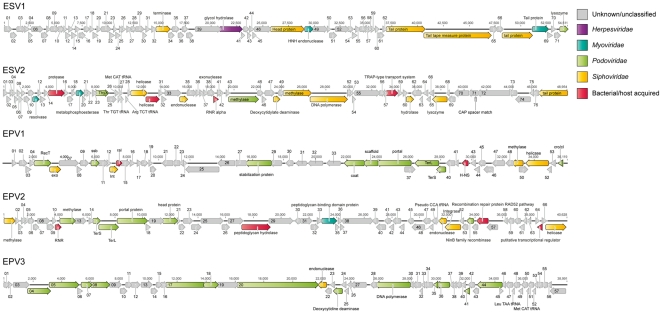
Genomic maps of the five largest genomes assembled from the phage metagenome (t_7_). Annotated open reading frames (ORFs) are colored based on the taxonomy of the top BLAST hit. ORFs labeled as bacterial/host acquired matched only to bacterial genes in the NCBI nr database, these genes may represent genes of prophage present in bacteria that have not been sampled in the viral refseq database.

Sequence similarity to reference phage genomes was used to classify the assembled EBPR phage, as three *Podoviridae* (EPV1–3) and six *Siphoviridae* (ESV1–6). EPV1 and EPV2 were inferred to be lysogenic, as they contain integrase proteins (Figure 1). Only EPV3 had detectable synteny to any previously sequenced phage (*Pseudomonas* phage 119X) (Figure S2). Further, only 30% of the t_7_ metagenome had a significant match (e-value≤10^−4^) in the NCBI viral refseq database, reflecting the general undersampling of environmental phage.

We investigated the presence of t_7_ phage in the t_0_ microbial metagenome and found that EPV1 was the only phage genome sampled at both time points. However, GAAS community analysis of the t_7_ metagenome showed that EPV1 was not the most abundant phage (Table 1). The high frequency of turnover in CAP populations and the high specificity of phage host range could account for such a large change in viral community composition over a relatively short time period [22]. Alternatively, this may be due to sampling bias in the microbial metagenome as phage were not specifically enriched at t_0_, contrary to the t_7_ sample. However, phage sequences have been detected in high numbers in bulk metagenomes not specifically enriched for virions [23], [24].

### Selective pressures on EBPR phage populations

**Figure 2:**
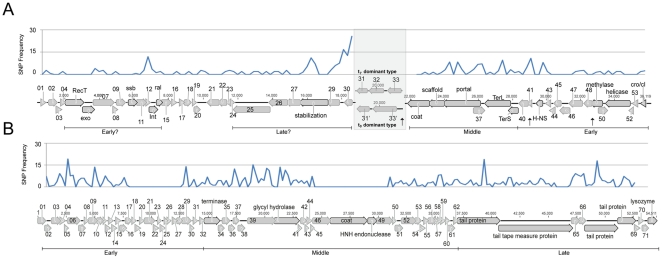
SNP frequencies between (A) ESV1 and (B) EPV1 genotypes. Frequencies were calculated using a sliding window and are expressed as a percentage of mismatching bases. The EPV1 genome is marked with a grey box showing the dominant genotype at each sampling point and the position of the H-NS gene is marked in bold. The position of the three H-NS binding sites in the EPV1 genome are marked with a black arrow underneath ORFs gp33, gp40 and gp48. The region of major divergence between the two EPV1 genotypes (shaded in grey) was not used in the calculation. Proposed transcriptional phases for each genome are labeled below as early, middle or late based on the presence of marker genes typically associated with the temporal classification of transcripts [55], [56]. A question mark indicates that the transcriptional phase was uncertain.

We investigated nucleotide variation within each t_7_ phage population to assess possible evolutionary pressures. Most populations were homogeneous with the exception of ESV1, which could be resolved into two distinct genotypes based on single nucleotide polymorphisms (SNP) patterns (Table 1; Figure 2). By reassembling reads from the t_0_ metagenome that mapped to EPV1, we identified a second genotype for this population only present at t_0_. The two EPV1 genotypes were significantly different in the region between the coat and stabilization genes where it appears that two hypothetical genes were replaced by three different hypothetical genes (Figure 2). Comparison of the ESV1 and EPV1 genotypes suggest similar selective pressures on these EBPR phage populations as the regions of highest variation in both populations were found in structural proteins such as tail or portal proteins (Figure 2). This is consistent with previous observations that phage structural proteins and their corresponding host receptors are in a constant state of co-evolution, an arms race, which can result in numerous new variants with altered outer membranes or coat proteins [25], [26]. The *d_N_/d_S_* ratios for ESV1 and EPV1 show that the great majority of their genes were under purifying selection (*d_N_/d_S_*<1, Table S2). Purifying selection has been observed in many viruses where there is strong selective pressure to maintain small genome sizes and resist random changes [27].

### Discovery of a bacterial regulatory gene in EPV1

A gene encoding a homolog of the global transcriptional regulator, heat-stable nucleoid structuring (H-NS) protein, was detected in the EPV1 population (both genotypes, Figure 2). Transcriptomic studies in enteric bacteria have shown that host-encoded H-NS down regulates expression of up to 5% of host genes [17]. Although H-NS has been found on plasmids [28], this is the first report of a H-NS gene in a phage genome. A search of public phage metagenomes from MG-RAST [29] CAMERA [30] and IMG-M [31] only identified H-NS in a small number of wastewater phage sequences. A functional H-NS requires two domains, a C-terminal DNA binding domain and an N-terminal oligomerization domain [32], [33]. Some phage carry the H-NS oligomerization domain that de-represses genes under host H-NS transcriptional control required for infection [34]. However, it is likely that the H-NS of EPV1 increases the repression of H-NS controlled genes as it contains both the oligomerization and DNA binding domains (Figure S3). When compared against the NCBI nr database, the EPV1 H-NS was most similar to a H-NS gene in the CAP genome (47% amino acid identity; Figure S3). This suggests that EPV1 laterally acquired its full length H-NS gene from CAP and that CAP is a host of this phage.

**Figure 3:**
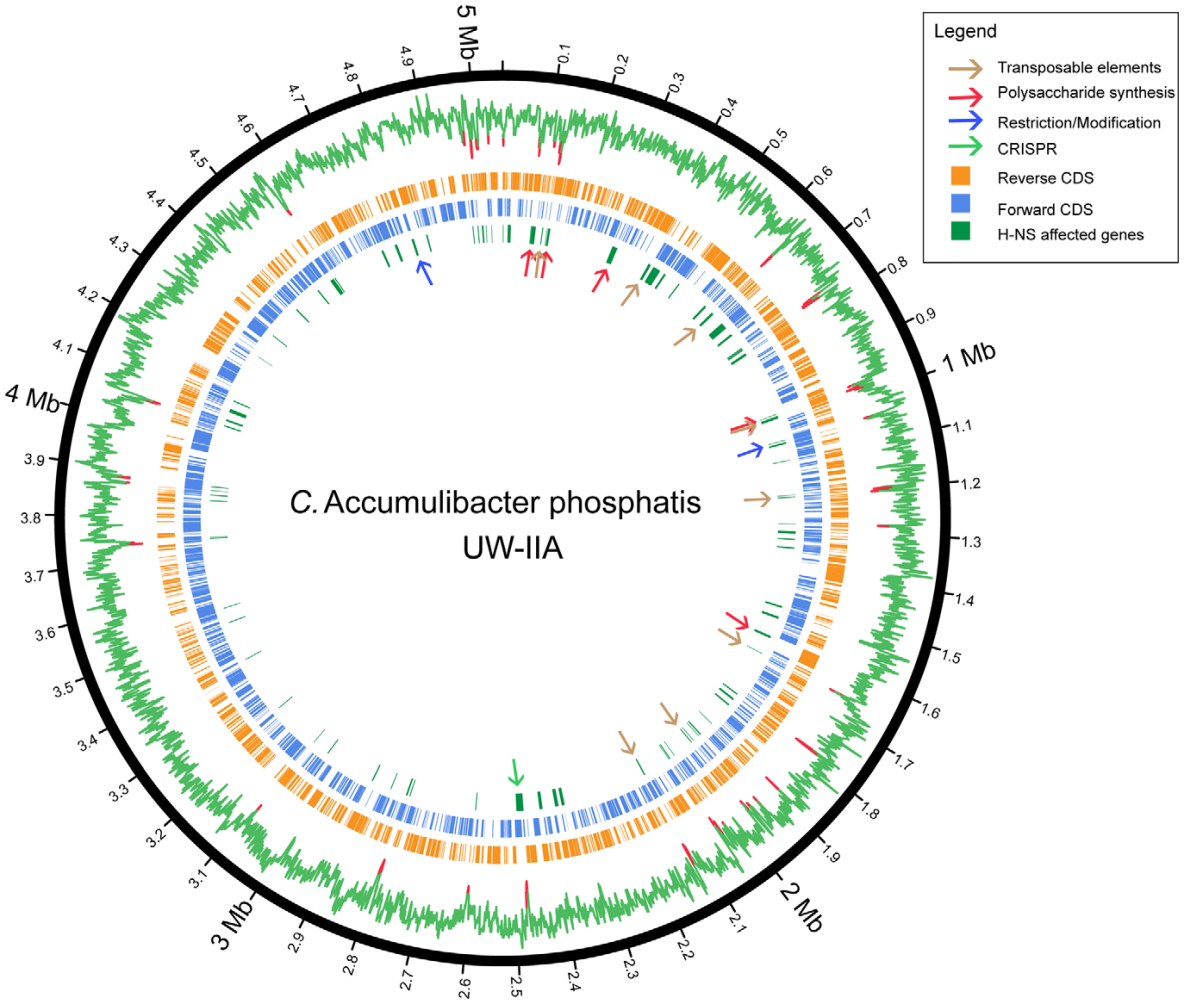
Location of putative H-NS regulated genes in the CAP genome. The GC-content of the CAP genome is represented by the outermost ring. Regions with less than 55% GC are highlighted in red. The genes on the posistive and negative strand of the CAP genome are represented by blue and orange rings, respectively. The innermost green ring marks the position of all the H-NS affected genes. The approximate positions of genes that may affect phage infection are highlighted by arrows in the inner most layer.

H-NS is a generic means for many bacterial species to down regulate newly acquired genes in hyper-variable regions of their genomes (e.g. transposons) [10]. H-NS binds preferentially to AT-rich sequences which are often characteristic of these dynamic regions [35]. To predict which genes are under H-NS control in CAP, putative binding sites were identified by comparative analysis with characterized H-NS binding profiles in related model proteobacteria (see methods). The predicted H-NS binding profile of CAP suggests that it too is able to repress hypervariable regions including polysaccharide biosynthesis gene cassettes, a Type III restriction-modification system and a CRISPR locus (Figure 3, Table S3). Notably, these are all key phage defense mechanisms.

Although the host-encoded H-NS in CAP has not been shown to repress CRISPR expression, this functionality has been demonstrated in *Enterobacteriaceae* [14], [15], [16], [36]. We propose that the H-NS of EPV1 can repress the CAP CRISPR and other key phage defense mechanisms, giving EPV1 a selective advantage over other phage when infecting CAP. Phage repression of host genes under H-NS regulation could only occur after infection and would therefore only be effective against intracellular defense mechanisms. Moreover, the phage H-NS would need to be expressed quickly in order to be effective. The location of the H-NS gene in the early expressed cohort of phage genes (Figure 2) is consistent with this hypothesis. Repression of CRISPR and restriction-modification systems would be highly beneficial to phage as they would not have to go through repeated rounds of evolution every time a new CRISPR spacer is introduced in the host genome. More generally, repression of up to 6% of CAP genes (Table S3) would be favorable to a phage by making available more resources (e.g. nucleotides, ATP) for virion synthesis. The potential for EPV1 to dramatically alter its host's gene expression to favor its own infection may help to explain the persistence of EPV1 in the EBPR bioreactor between t_0_ and t_7_ sampling. Three putative H-NS binding sites were also identified in the EPV1 genome adjacent to genes gp33, gp40, and gp48, the first of which falls in the variable region between the two EPV1 strains (Figure 2). These may play a role in controlling the expression of phage genes and associated virion production.

Phage are increasingly being recognized for their ability to manipulate their hosts using host-acquired genes. This has mostly been via up-regulation of key metabolic genes such as photosystem (psbA) and phosphate metabolic (phoH) genes in cyanobacteria [37]. By comparison, the interaction mediated by a phage-encoded H-NS would manipulate the host genome via down-regulation of many genes. H-NS is widely distributed in the Proteobacteria, particularly the Beta- and Gammaproteobacteria [38] (Figure S4). We speculate that ecosystems dominated by members of these groups, such as EBPR, will harbor and be susceptible to phage that carry host-derived H-NS. This previously unrecognized Achilles heel in bacterial defense systems may have significant implications in the phage-host arms race and warrants further investigation.

## Methods

### Microbial and phage metagenomic datasets

Two previously published metagenomic datasets obtained from the same lab-scale EBPR reactor operated in Madison, WI, USA, were used in the present study. The first (herein, t_0_) was a bulk microbial metagenome (acc. no. AATO00000000) leading to the reconstruction of the dominant bacterial population in the bioreactor, *Candidatus* Accumulibacter phosphatis, Type IIA [6]. The second dataset, sampled seven months later (herein t_7_), was obtained from phage as previously described [20]. Briefly, phage virions were purified using density gradient cesium chloride ultracentrifugation, and a linker-amplified shotgun library was constructed [20]. Sanger sequencing of 16,807 clones from a single end using the AmpL1 primer produced ∼16 Mb of sequence data.

### Phage metagenome assembly

The t_7_ phage metagenomic data was quality trimmed using a Phred [39], [40] Q20 score and the reads were assembled using Velvet 1.0.05 [41] with a K-mer length of 37, expected coverage of 25 and a coverage cutoff of 2, which produced assemblies containing the longest contigs. CAP3 [42] was used to complement and confirm this assembly using an overlap of 35 bp with 85% identity. The Velvet and CAP3 assemblies were validated by tetranucleotide clustering [43], BLASTn [44] comparisons and manual inspection of contigs using Geneious 5.0.4 [45]. The largest contigs from each assembly were compared against each other with BLASTn to determine if one assembler had broken a contig into smaller parts. These breaks or inconsistencies were checked manually to determine whether this was due to misassembly. Tetranucleotide frequencies were generated for individual contigs and all contigs over 2 kb in size were subjected to tetranucleotide clustering using a k-means algorithm with a max of 20 iterations. Contig clustering allowed for multiple contigs to be assigned to a single phage genome. Assembled contigs belonging to the nine dominant phage have been deposited to the public databases under accession numbers JF412294-JF412302.

### Annotation of phage contigs

Open Reading Frames (ORFs) were predicted using FGENESB (www.softberry.com) to call the position of the ORFs. Each ORF was extracted and compared against the NCBI non-redundant protein database (nr) using BLASTp. Each ORF that returned a match with e-value<10^−3^ was manually examined for potential function through homology to the most significant BLASTp similarity. The phylogeny of each annotated gene was determined by comparing them to all other virus genomes with BLASTx and assigning the phylogeny based on the highest similarity match with an e-value cutoff of 10^−3^. Contigs were then assigned an overall phylogeny by comparing the number of genes that fell into each recognized family of phage.

### Analysis of phage contigs

Phage contigs were compared to the published EBPR microbial metagenome (acc. AATO00000000) [6] using BLASTn to determine whether any phage were also sampled at that time point. Contigs with alignment lengths greater than 5 kb were compared against each other using both Dotmatcher (http://emboss.sourceforge.net) (sliding window and probabilistic scoring matrix window size: 100, threshold: 75 and tile size: 10000) and the Mauve genome aligner [46] (mauveAligner algorithm, match seed weight: 15, minimum LCB score: 69, using MUSCLE3.6 for the gapped alignment algorithm). Strain determination was performed using Strainer 1.0 [47]. MEGA 5 [48] was used to calculate d_N_/d_S_ ratios and the SNP frequency between strains was calculated for 250 bp windows across genotypes. Phage contigs from the t_0_ metagenome were reassembled by mapping the entire t_0_ metagenome onto the contigs of the t_7_ phage genomes. The subset of reads that mapped to each contig were independently reassembled using CAP3.

### Community structure and diversity analysis

The community structure of the t_7_ metagenome was analysed using PHACCS [21] and GAAS 0.15 [49] to generate estimates of the community composition. PHACCS estimates of richness the average genome size calculated by GAAS 0.15 and a contig spectrum generated by Circonspect 0.2.4 (10× coverage), using the Minimo assembler and default parameters. Furthermore GAAS was used to generate estimates of the community composition using parameters of 50% identity, 50% read coverage, minimum 10e^−6^ e-value, weighing all hits and normalizing for genome length. When t_7_ was compared solely to the NCBI refseq database only 1.5% of the metagenomic reads had similarities. However, by adding in the nine phage genomes from the assembly over 66% of the reads matched.

### Heat-stable Nucleoid-Structuring (H-NS) gene analysis

A homolog of the H-NS gene found in the genome of EPV1 was aligned with H-NS homologs from the alpha-, beta- and gamma- subdivisions of the proteobacteria using ClustalW 2.0.11 [50]. A phylogenetic tree was made with ARB [51] using the maximum likelihood method RAxML and the Dayhoff substitution model. A genome wide scan of CAP for H-NS binding sites was performed by a pattern search, using the high affinity site of *E. coli* K12 [52], with a maximum of two mismatches. Over 1,000 sites were found and these were analyzed using Weblogo (http://weblogo.berkeley.edu/logo.cgi) to generate the CAP high affinity site. This new pattern, TCGANNAATT, was used to search for CAP and EBPR phage genes and operons that may be affected by H-NS. Since H-NS is strongly associated with regions of low GC-content [53], [54], operons associated with 1 kb regions that had a GC-content of less than 55% were combined with those found by the pattern matching.

## Supporting Information

Figure S1 **Tetranucleotide clustering of contigs from the nine phage genomes.** (A) Clustering of all contigs over 2 kb using tetranucleotide binning using a window size of 2 kb. The k-mer frequencies for each 2 kb window (rows) were calculated and grouped using k-means clustering and displayed in a heatmap of low frequency (black) to high frequency (white) for each individual tetranucleotide combination (columns). The metagenome can be grouped into three clusters: cluster 1, which has mid-range frequencies for both AT and GC rich tetranucleotides; cluster 2, which favors GC rich tetranucleotides; and cluster 3, which favors AT rich tetranucleotides. (B) Tetranucleotide signatures of the nine phage genomes. ESV1 was grouped into cluster 1; EPV1, EPV2, ESV2 – ESV6 were grouped into cluster 2; EPV3 was grouped into cluster 3.
(TIF)

Figure S2 **Synteny between the genomes of 119X and EPV3.** Orthologous genes between EPV3 and 119X are linked using colored quadrangles to indicate the BLASTx e-value and labeled with amino acid percent identity.
(TIF)

Figure S3 **Amino acid alignment of H-NS genes from EPV1 and 18 beta-proteobacteia.** Colored residues are conserved in greater than 50% of the sequences. Residues identified in *E. coli* K12 as being important to H-NS function are marked with a star (*).
(TIF)

Figure S4 **Phylogenetic relationship between H-NS homologs.** H-NS of EPV1 was aligned to homologs from the Alpha-, Beta-, and Gammaproteobacteria using maximum likelihood. The H-NS family member MvaT from *Pseudomonas fluorescens* was used as an outgroup.
(TIF)

Table S1 **Assembly statistics for Velvet and CAP3.**
(DOC)

Table S2 **Genetic position and d_N_/d_S_ ratio of genes from the ESV1 and EPV1 phage genomes.** Only ORFs where the entire coding length feel inside regions of genetic variation. The ESV1 calculations are based on the two genotypes found in t_7_; EPV1 calculations are for the two genotypes found in t_0_
(DOC)

Table S3 **CAP genes that were identified as being under potential H-NS regulation.**
(DOC)
