# Supplementary figures and images for: Phage Encoded H-NS: A Potential Achilles Heel in the Bacterial Defence System
Source: PLoS One. 2011 May 18;6(5):e20095. doi: 10.1371/journal.pone.0020095 (PMC3097231; doi:10.1371/journal.pone.0020095)

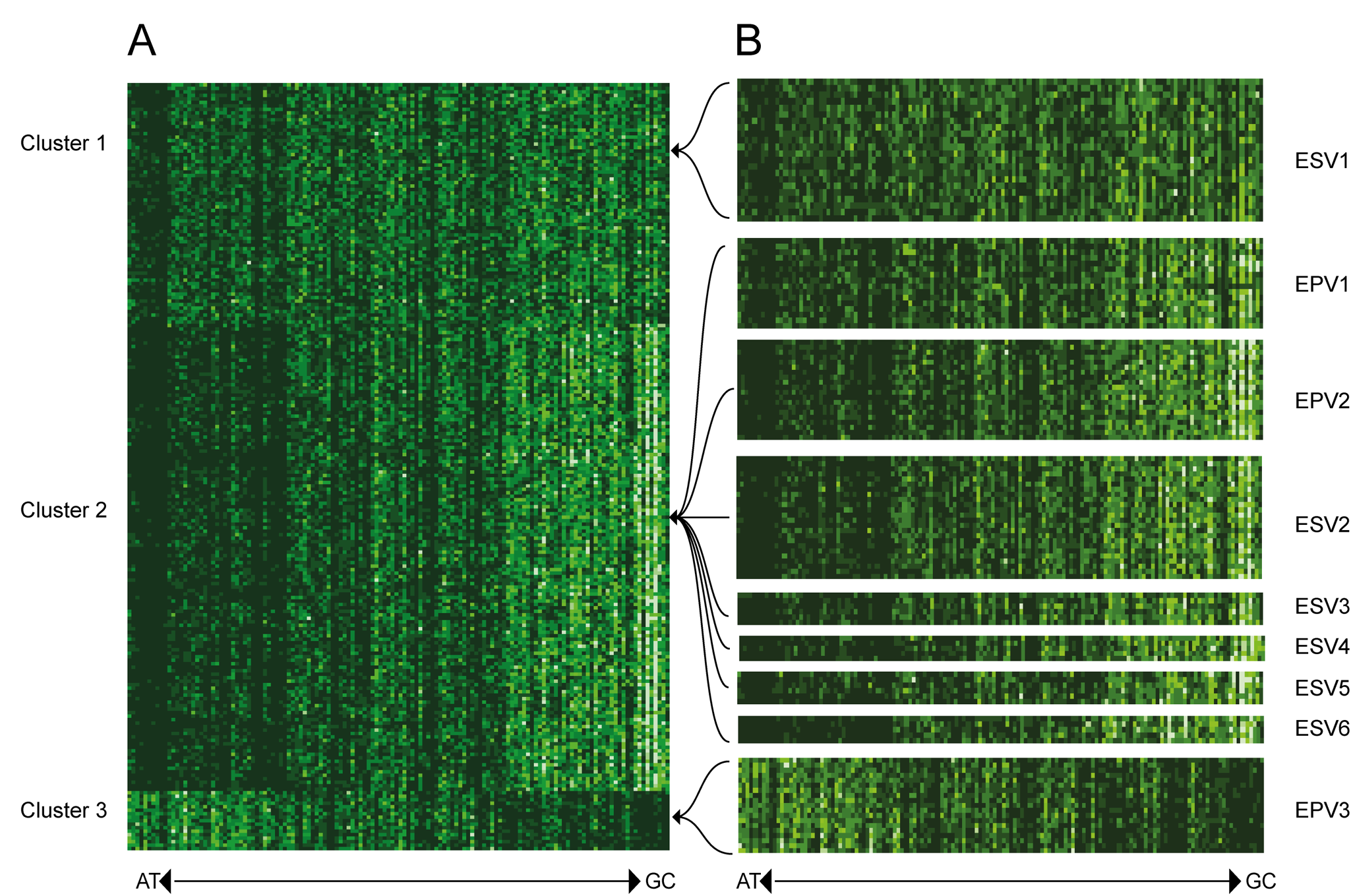

Supplement: Figure S1 — Tetranucleotide clustering of contigs from the nine phage genomes. (A) Clustering of all contigs over 2 kb using tetranucleotide binning using a window size of 2 kb. The k-mer frequencies for each 2 kb window (rows) were calculated and grouped using k-means clustering and displayed in a heatmap of low frequency (black) to high frequency (white) for each individual tetranucleotide combination (columns). The metagenome can be grouped into three clusters: cluster 1, which has mid-range frequencies for both AT and GC rich tetranucleotides; cluster 2, which favors GC rich tetranucleotides; and cluster 3, which favors AT rich tetranucleotides. (B) Tetranucleotide signatures of the nine phage genomes. ESV1 was grouped into cluster 1; EPV1, EPV2, ESV2 – ESV6 were grouped into cluster 2; EPV3 was grouped into cluster 3. (TIF) [file pone.0020095.s001.tif]

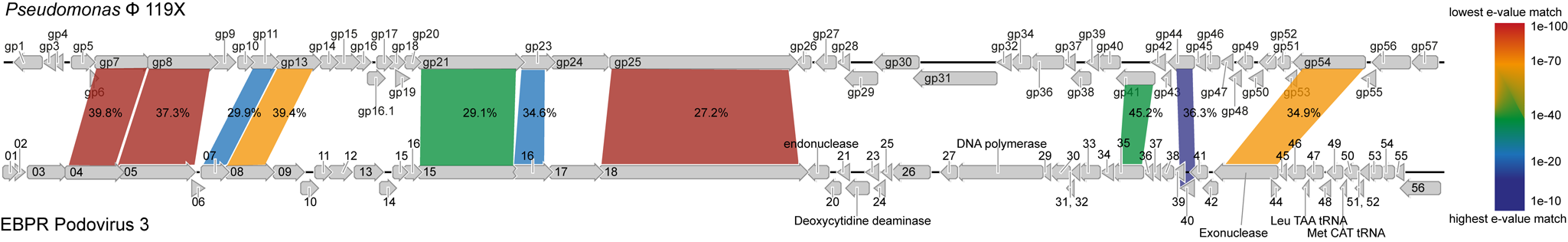

Supplement: Figure S2 — Synteny between the genomes of 119X and EPV3. Orthologous genes between EPV3 and 119X are linked using colored quadrangles to indicate the BLASTx e-value and labeled with amino acid percent identity. (TIF) [file pone.0020095.s002.tif]

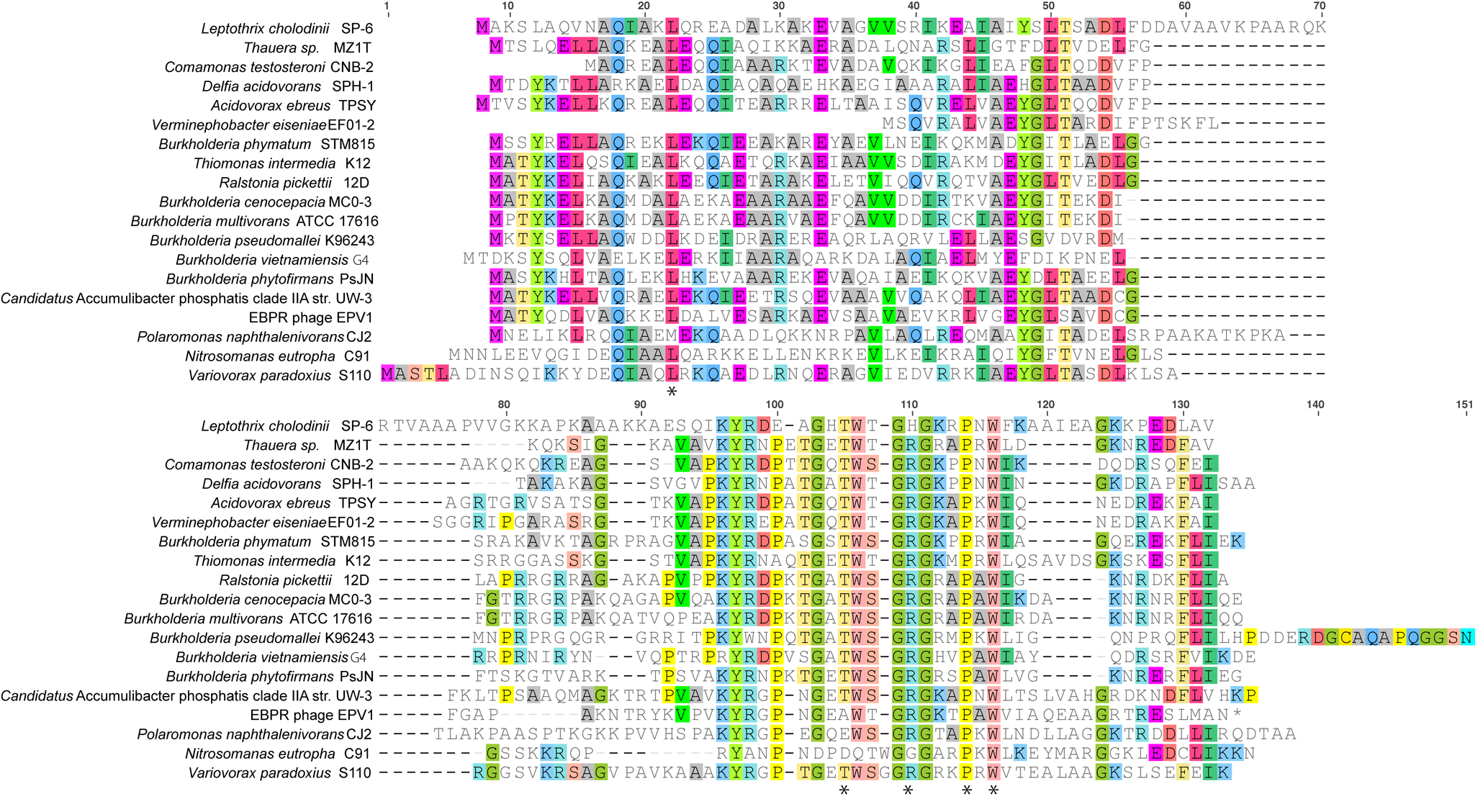

Supplement: Figure S3 — Amino acid alignment of H-NS genes from EPV1 and 18 beta-proteobacteia. Colored residues are conserved in greater than 50% of the sequences. Residues identified in E. coli K12 as being important to H-NS function are marked with a star (*). (TIF) [file pone.0020095.s003.tif]

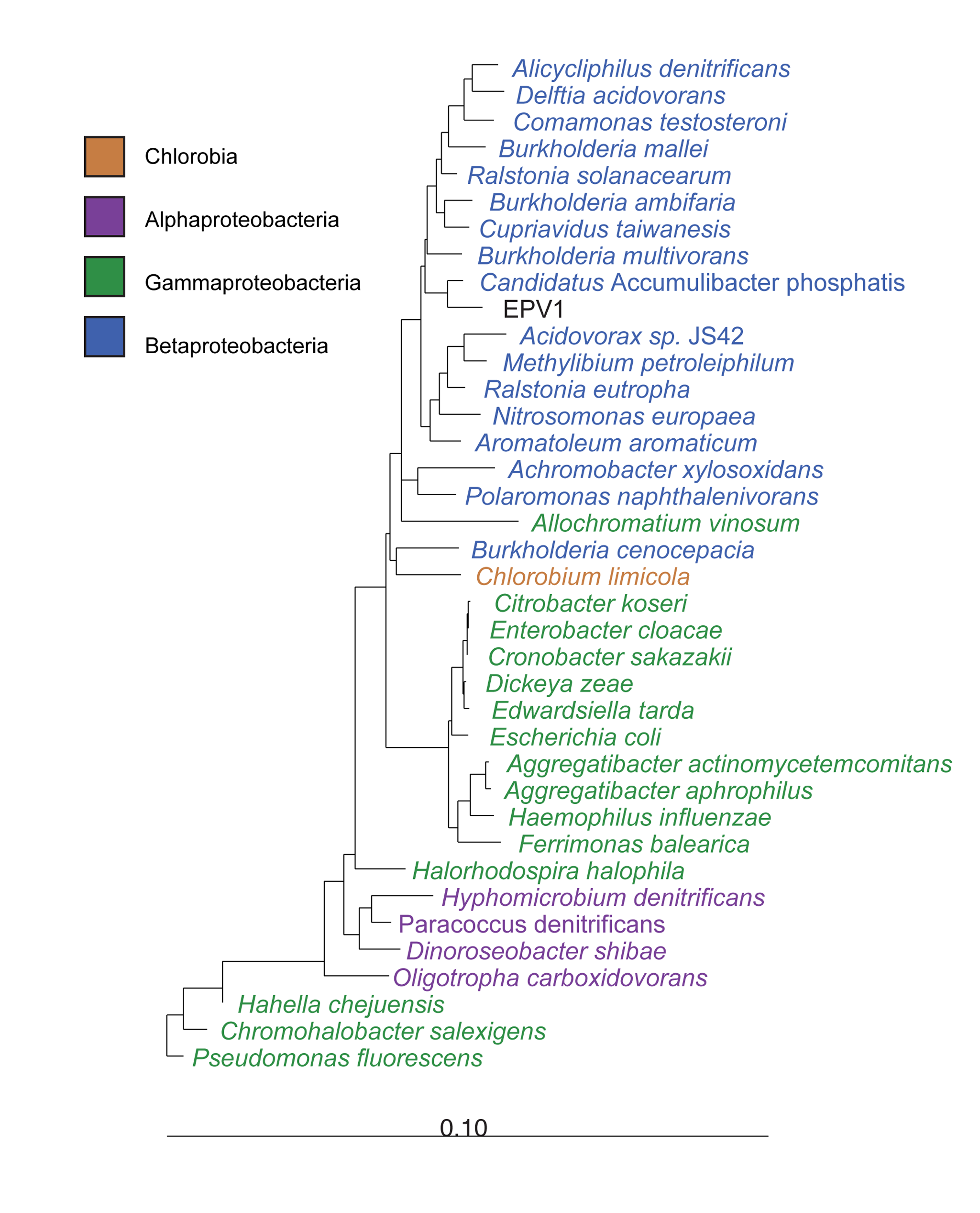

Supplement: Figure S4 — Phylogenetic relationship between H-NS homologs. H-NS of EPV1 was aligned to homologs from the Alpha-, Beta-, and Gammaproteobacteria using maximum likelihood. The H-NS family member MvaT from Pseudomonas fluorescens was used as an outgroup. (TIF) [file pone.0020095.s004.tif]
