# Supplementary material for: Phage Encoded H-NS: A Potential Achilles Heel in the Bacterial Defence System
Source: PLoS One. 2011 May 18;6(5):e20095. doi: 10.1371/journal.pone.0020095 (PMC3097231; doi:10.1371/journal.pone.0020095)
Supplement: Table S1 — Assembly statistics for Velvet and CAP3. (DOC) [file pone.0020095.s005.doc]

|  | Velvet | CAP3 |
| --- | --- | --- |
| Number of contigs | 803 | 691 |
| Total length of contigs | 815, 631 bp | 1, 306, 805 bp |
| Number of reads in contigs | 12, 029 | 13, 077 |
| Maximum length of contig | 48, 918 bp | 41, 199 bp |
| N50 | 507 | 945 |
