# Supplementary material for: Phage Encoded H-NS: A Potential Achilles Heel in the Bacterial Defence System
Source: PLoS One. 2011 May 18;6(5):e20095. doi: 10.1371/journal.pone.0020095 (PMC3097231; doi:10.1371/journal.pone.0020095)
Supplement: Table S2 — Genetic position and dN/dS ratio of genes from the ESV1 and EPV1 phage genomes. Only ORFs where the entire coding length feel inside regions of genetic variation. The ESV1 calculations are based on the two genotypes found in t7; EPV1 calculations are for the two genotypes found in t0 (DOC) [file pone.0020095.s006.doc]

| **ESV1** |  |  |  |
| --- | --- | --- | --- |
| **Gene** | **Start** | **End** | **d_N_/d_S_ ratio** |
| gp02 | 638 | 1105 | 0.545454545 |
| gp04 | 1982 | 2395 | 0.619469027 |
| gp10 | 5245 | 5739 | 0.04587156 |
| gp15 | 6889 | 7275 | 0.142857143 |
| terminase | 14720 | 15967 | 0.158730159 |
| gp35 | 16309 | 16866 | 0.580645161 |
| gp36 | 16863 | 17279 | 0.2 |
| gp41 | 23001 | 23213 | 0.203703704 |
| gp42 | 23210 | 23611 | 0.067961165 |
| gp43 | 23608 | 23808 | 0.375 |
| gp44 | 23775 | 24140 | 0.289473684 |
| gp45 | 24094 | 24291 | 0.909090909 |
| gp52 | 32092 | 33273 | 0.060606061 |
| gp54 | 33725 | 34075 | 0.048780488 |
| gp55 | 34062 | 34412 | 0.163793103 |
| gp56 | 34431 | 35027 | 0.192307692 |
| gp58 | 35251 | 35544 | 0 |
| gp59 | 35547 | 35861 | 0.090163934 |
| gp62 | 36695 | 36994 | 0.16017316 |
| minor tail protein | 37024 | 40659 | 0.053571429 |
| Tail tape measure protein | 40656 | 47081 | 0 |
| gp69 | 52621 | 53019 | 0.578947368 |
| **EPV1** |  |  |  |
| stabilization | 15549 | 17123 | 0.096 |
| gp29 | 17134 | 17790 | 2 |
| coat | 21842 | 23056 | 0.2222 |
| scafold | 23083 | 23916 | 0.1755 |
| portal | 23931 | 26042 | 0.725 |
| gp41 | 28368 | 28838 | 1.63 |
