# Supplementary material for: Phage Encoded H-NS: A Potential Achilles Heel in the Bacterial Defence System
Source: PLoS One. 2011 May 18;6(5):e20095. doi: 10.1371/journal.pone.0020095 (PMC3097231; doi:10.1371/journal.pone.0020095)
Supplement: Table S3 — CAP genes that were identified as being under potential H-NS regulation. (DOC) [file pone.0020095.s007.doc]

| Gene | Start | End | Product |
| --- | --- | --- | --- |
| CAP2UW1_0015 | 14455 | 15138 | Lytic transglycosylase catalytic |
| CAP2UW1_0016 | 15192 | 17663 | glycerol-3-phosphate acyltransferase |
| CAP2UW1_0017 | 17696 | 19744 | elongation factor G |
| CAP2UW1_0018 | 19993 | 22290 | malic enzyme |
| CAP2UW1_0069 | 80182 | 81180 | glycosyl transferase family 2 |
| CAP2UW1_0070 | 81177 | 82502 | polysaccharide biosynthesis protein |
| CAP2UW1_0071 | 82499 | 84817 | Spermidine synthase-like protein |
| CAP2UW1_0072 | 84825 | 86879 | glutamine amidotransferase class-II |
| CAP2UW1_0073 | 86876 | 88057 | hypothetical protein |
| CAP2UW1_0074 | 88054 | 91224 | hypothetical protein |
| CAP2UW1_0106 | 126411 | 127658 | glycosyl transferase group 1 |
| CAP2UW1_0107 | 127860 | 129017 | LPS biosynthesis protein WbpG |
| CAP2UW1_0108 | 129014 | 129628 | imidazole glycerol phosphate synthase subunit HisH |
| CAP2UW1_0109 | 129630 | 130427 | imidazole glycerol phosphate synthase subunit HisF |
| CAP2UW1_0110 | 130438 | 131466 | polysaccharide biosynthesis protein CapD |
| CAP2UW1_0254 | 309927 | 310892 | glucokinase |
| CAP2UW1_0255 | 310915 | 313368 | glycogen/starch/alpha-glucan phosphorylase |
| CAP2UW1_0256 | 313380 | 315563 | 1,4-alpha-glucan branching enzyme |
| CAP2UW1_0257 | 315551 | 317566 | glycogen debranching enzyme GlgX |
| CAP2UW1_0258 | 317608 | 318576 | hypothetical protein |
| CAP2UW1_0259 | 318614 | 319579 | protein of unknown function DUF815 |
| CAP2UW1_0260 | 319629 | 320879 | MltA domain protein |
| CAP2UW1_0261 | 321055 | 322257 | Rh family protein/ammonium transporter |
| CAP2UW1_0262 | 322363 | 323874 | threonine dehydratase |
| CAP2UW1_0351 | 419877 | 421403 | Integrase catalytic region |
| CAP2UW1_0352 | 421459 | 422262 | IstB domain protein ATP-binding protein |
| CAP2UW1_0353 | 424801 | 426315 | protein of unknown function DUF1214 |
| CAP2UW1_0364 | 435477 | 440702 | ATP-dependent DNA helicase, RecQ family |
| CAP2UW1_0365 | 440725 | 441444 | 3-ketoacyl-(acyl-carrier-protein) reductase |
| CAP2UW1_0366 | 441441 | 441893 | hypothetical protein |
| CAP2UW1_0367 | 441887 | 442690 | hypothetical protein |
| CAP2UW1_0368 | 442687 | 443880 | 3-oxoacyl-(acyl carrier protein) synthase I |
| CAP2UW1_0369 | 443966 | 444703 | Methyltransferase type 12 |
| CAP2UW1_0370 | 444700 | 445500 | polysaccharide deacetylase |
| CAP2UW1_0371 | 445506 | 446153 | hypothetical protein |
| CAP2UW1_0372 | 446150 | 448519 | putative transmembrane protein |
| CAP2UW1_0373 | 448516 | 449082 | hypothetical protein |
| CAP2UW1_0374 | 449079 | 449993 | lipid A biosynthesis acyltransferase |
| CAP2UW1_0375 | 449990 | 450292 | hypothetical protein |
| CAP2UW1_0376 | 450279 | 451634 | AMP-dependent synthetase and ligase |
| CAP2UW1_0377 | 451631 | 452302 | hypothetical protein |
| CAP2UW1_0378 | 452299 | 452583 | acyl carrier protein |
| CAP2UW1_0379 | 452602 | 453855 | Beta-ketoacyl synthase |
| CAP2UW1_0380 | 453859 | 454098 | phosphopantetheine-binding |
| CAP2UW1_0381 | 454285 | 455823 | integral membrane protein MviN |
| CAP2UW1_0382 | 455843 | 456472 | PEBP family protein |
| CAP2UW1_0398 | 470091 | 470465 | hypothetical protein |
| CAP2UW1_0399 | 470465 | 471361 | hypothetical protein |
| CAP2UW1_0400 | 471431 | 472621 | amidohydrolase |
| CAP2UW1_0401 | 472964 | 473644 | two component transcriptional regulator, winged helix family |
| CAP2UW1_0445 | 518497 | 519474 | cysteine synthase A |
| CAP2UW1_0446 | 519788 | 520579 | short-chain dehydrogenase/reductase SDR |
| CAP2UW1_0447 | 520612 | 521028 | hypothetical protein |
| CAP2UW1_0506 | 611819 | 613192 | cysteinyl-tRNA synthetase |
| CAP2UW1_0507 | 613301 | 614293 | TPR repeat-containing protein |
| CAP2UW1_0508 | 614354 | 614944 | Peptidylprolyl isomerase |
| CAP2UW1_0509 | 614941 | 615432 | peptidyl-prolyl cis-trans isomerase cyclophilin type |
| CAP2UW1_0510 | 615494 | 617308 | ATP-dependent DNA helicase RecQ |
| CAP2UW1_0527 | 639402 | 640187 | transposase/IS protein |
| CAP2UW1_0528 | 640184 | 641272 | Integrase catalytic region |
| CAP2UW1_0530 | 642270 | 643985 | Ribonuclease H |
| CAP2UW1_0531 | 644255 | 645550 | transposase IS204/IS1001/IS1096/IS1165 family protein |
| CAP2UW1_0565 | 679682 | 680611 | UDP-3-O-[3-hydroxymyristoyl] N-acetylglucosamine deacetylase |
| CAP2UW1_0566 | 680707 | 681882 | cell division protein FtsZ |
| CAP2UW1_0567 | 681951 | 683180 | cell division protein FtsA |
| CAP2UW1_0568 | 683177 | 683929 | Polypeptide-transport-associated domain protein FtsQ-type |
| CAP2UW1_0569 | 683933 | 684859 | D-alanine--D-alanine ligase |
| CAP2UW1_0570 | 684869 | 686257 | UDP-N-acetylmuramate/alanine ligase |
| CAP2UW1_0571 | 686254 | 687312 | undecaprenyldiphospho-muramoylpentapeptide beta-N- acetylglucosaminyltransferase |
| CAP2UW1_0572 | 687365 | 688525 | cell division protein FtsW |
| CAP2UW1_0573 | 688525 | 689892 | UDP-N-acetylmuramoylalanine/D-glutamate ligase |
| CAP2UW1_0574 | 689894 | 690982 | phospho-N-acetylmuramoyl-pentapeptide- transferase |
| CAP2UW1_0575 | 690982 | 692352 | UDP-N-acetylmuramoylalanyl-D-glutamyl-2, 6-diaminopimelate/D-alanyl-D-alanyl ligase |
| CAP2UW1_0576 | 692361 | 693869 | UDP-N-acetylmuramyl-tripeptide synthetase |
| CAP2UW1_0577 | 693866 | 695617 | Peptidoglycan glycosyltransferase |
| CAP2UW1_0578 | 695614 | 695892 | cell division protein FtsL |
| CAP2UW1_0579 | 695894 | 696844 | S-adenosyl-methyltransferase MraW |
| CAP2UW1_0580 | 696841 | 697287 | cell division protein MraZ |
| CAP2UW1_0603 | 718434 | 718907 | Glyoxalase/bleomycin resistance protein/dioxygenase |
| CAP2UW1_0604 | 718915 | 719049 | hypothetical protein |
| CAP2UW1_0605 | 719102 | 719434 | Thioredoxin domain protein |
| CAP2UW1_0606 | 719451 | 720617 | efflux transporter, RND family, MFP subunit |
| CAP2UW1_0607 | 720627 | 723719 | acriflavin resistance protein |
| CAP2UW1_0648 | 766354 | 766650 | hypothetical protein |
| CAP2UW1_0650 | 769008 | 769769 | hypothetical protein |
| CAP2UW1_0652 | 772363 | 772944 | hypothetical protein |
| CAP2UW1_0653 | 773034 | 773444 | glutathione-dependent formaldehyde-activating GFA |
| CAP2UW1_0664 | 785721 | 787493 | bifunctional isocitrate dehydrogenase kinase/phosphatase protein |
| CAP2UW1_0665 | 787490 | 788416 | SdiA-regulated domain protein |
| CAP2UW1_0666 | 788462 | 789268 | hydroxypyruvate isomerase |
| CAP2UW1_0667 | 789318 | 789878 | ATP/cobalamin adenosyltransferase |
| CAP2UW1_0668 | 789921 | 790478 | hypothetical protein |
| CAP2UW1_0847 | 974295 | 974879 | transferase hexapeptide repeat containing protein |
| CAP2UW1_0853 | 981159 | 982484 | transposase IS4 family protein |
| CAP2UW1_0854 | 982635 | 983459 | hypothetical protein |
| CAP2UW1_0855 | 983481 | 984641 | glycosyl transferase group 1 |
| CAP2UW1_0909 | 1050779 | 1050880 | adenine specific DNA-methyltransferase |
| CAP2UW1_0912 | 1053450 | 1053986 | hypothetical protein |
| CAP2UW1_0913 | 1053979 | 1054695 | putative two-component response-regulatory protein YehT |
| CAP2UW1_0914 | 1054689 | 1056395 | signal transduction histidine kinase, LytS |
| CAP2UW1_0915 | 1056471 | 1057730 | hypothetical protein |
| CAP2UW1_0960 | 1103710 | 1104264 | putative cache sensor protein |
| CAP2UW1_0961 | 1104357 | 1104920 | GPR1/FUN34/yaaH family protein |
| CAP2UW1_1046 | 1200322 | 1200630 | hypothetical protein |
| CAP2UW1_1051 | 1205418 | 1205528 | hypothetical protein |
| CAP2UW1_1053 | 1206227 | 1206595 | hypothetical protein |
| CAP2UW1_1121 | 1281125 | 1281418 | hypothetical protein |
| CAP2UW1_1135 | 1301051 | 1301176 | hypothetical protein |
| CAP2UW1_1136 | 1301173 | 1303161 | acetyl-CoA carboxylase, biotin carboxylase |
| CAP2UW1_1137 | 1303181 | 1304713 | carboxyl transferase |
| CAP2UW1_1138 | 1304787 | 1305785 | arginine/ornithine transport system ATPase |
| CAP2UW1_1139 | 1305819 | 1307993 | methylmalonyl-CoA mutase |
| CAP2UW1_1153 | 1322440 | 1323228 | Indole-3-glycerol-phosphate synthase |
| CAP2UW1_1154 | 1323256 | 1324281 | anthranilate phosphoribosyltransferase |
| CAP2UW1_1155 | 1324278 | 1324850 | glutamine amidotransferase of anthranilate synthase |
| CAP2UW1_1178 | 1346143 | 1346742 | putative signal transduction protein with CBS domains |
| CAP2UW1_1179 | 1346774 | 1348339 | Gamma-glutamyltransferase |
| CAP2UW1_1180 | 1348433 | 1349530 | response regulator receiver modulated CheB methylesterase |
| CAP2UW1_1334 | 1511073 | 1512203 | hypothetical protein |
| CAP2UW1_1335 | 1512289 | 1513791 | hypothetical protein |
| CAP2UW1_1336 | 1513775 | 1515367 | hypothetical protein |
| CAP2UW1_1357 | 1541273 | 1542610 | hypothetical protein |
| CAP2UW1_1358 | 1542615 | 1542905 | hypothetical protein |
| CAP2UW1_1359 | 1542902 | 1543315 | flagellar protein FliS |
| CAP2UW1_1360 | 1543325 | 1544734 | flagellar hook-associated 2 domain protein |
| CAP2UW1_1361 | 1544785 | 1545150 | flagellar protein FlaG protein |
| CAP2UW1_1362 | 1545244 | 1547040 | flagellin domain protein |
| CAP2UW1_1414 | 1598656 | 1600119 | PEP-CTERM system associated protein |
| CAP2UW1_1415 | 1600129 | 1601220 | secretion ATPase, PEP-CTERM locus subfamily |
| CAP2UW1_1416 | 1601233 | 1602414 | UDP-N-acetylglucosamine 2-epimerase |
| CAP2UW1_1417 | 1602423 | 1603289 | polysaccharide deactylase family protein, PEP-CTERM locus subfamily |
| CAP2UW1_1418 | 1603286 | 1604371 | FemAB-related protein, PEP-CTERM system-associated |
| CAP2UW1_1589 | 1798727 | 1799446 | hypothetical protein |
| CAP2UW1_1590 | 1799518 | 1803465 | glycogen debranching enzyme GlgX |
| CAP2UW1_1603 | 1819041 | 1820270 | hypothetical protein |
| CAP2UW1_1604 | 1820320 | 1820775 | hypothetical protein |
| CAP2UW1_1605 | 1820833 | 1821624 | hypothetical protein |
| CAP2UW1_1606 | 1821633 | 1822625 | alpha/beta hydrolase fold protein |
| CAP2UW1_1707 | 1931746 | 1934502 | hypothetical protein |
| CAP2UW1_1710 | 1939058 | 1939741 | hypothetical protein |
| CAP2UW1_1740 | 1965333 | 1965941 | hypothetical protein |
| CAP2UW1_1817 | 2039340 | 2039450 | hypothetical protein |
| CAP2UW1_1820 | 2043144 | 2043830 | hypothetical protein |
| CAP2UW1_1883 | 2122571 | 2123533 | 5'-nucleotidase |
| CAP2UW1_2092 | 2357649 | 2358425 | hypothetical protein |
| CAP2UW1_2093 | 2358562 | 2359242 | hypothetical protein |
| CAP2UW1_2094 | 2359531 | 2361732 | sulphate transporter |
| CAP2UW1_2095 | 2361729 | 2361830 | hypothetical protein |
| CAP2UW1_2096 | 2361970 | 2362476 | UspA domain protein |
| CAP2UW1_2097 | 2362586 | 2363203 | HhH-GPD family protein |
| CAP2UW1_2098 | 2363303 | 2364271 | acetyl-CoA carboxylase carboxyltransferase subunit alpha |
| CAP2UW1_2099 | 2364240 | 2365646 | tRNA(Ile)-lysidine synthetase |
| CAP2UW1_2107 | 2374876 | 2375304 | Nucleoside-diphosphate kinase |
| CAP2UW1_2108 | 2375324 | 2376445 | radical SAM enzyme, Cfr family |
| CAP2UW1_2109 | 2376442 | 2377245 | type IV pilus biogenesis/stability protein PilW |
| CAP2UW1_2110 | 2377301 | 2378179 | hypothetical protein |
| CAP2UW1_2111 | 2378184 | 2379449 | 4-hydroxy-3-methylbut-2-en-1-yl diphosphate synthase |
| CAP2UW1_2112 | 2379446 | 2380741 | histidyl-tRNA synthetase |
| CAP2UW1_2113 | 2380744 | 2381427 | hypothetical protein |
| CAP2UW1_2114 | 2381427 | 2382569 | outer membrane assembly lipoprotein YfgL |
| CAP2UW1_2115 | 2382566 | 2384044 | GTP-binding protein EngA |
| CAP2UW1_2149 | 2419556 | 2424862 | DEAD/DEAH box helicase domain protein |
| CAP2UW1_2150 | 2424872 | 2427712 | helicase domain protein |
| CAP2UW1_2192 | 2472216 | 2472521 | CRISPR-associated protein Cas2 |
| CAP2UW1_2193 | 2472511 | 2473530 | CRISPR-associated protein Cas1 |
| CAP2UW1_2194 | 2473547 | 2473834 | CRISPR-associated protein Cas2 |
| CAP2UW1_2195 | 2474003 | 2474275 | hypothetical protein |
| CAP2UW1_2196 | 2474672 | 2475139 | hypothetical protein |
| CAP2UW1_2197 | 2479959 | 2481065 | hypothetical protein |
| CAP2UW1_2198 | 2481084 | 2482250 | CRISPR-associated protein, TM1812 family |
| CAP2UW1_2199 | 2482336 | 2483499 | CRISPR-associated RAMP protein, Cmr6 family |
| CAP2UW1_2200 | 2483496 | 2483858 | CRISPR-associated protein, Cmr5 family |
| CAP2UW1_2201 | 2483855 | 2484772 | CRISPR-associated RAMP protein, Cmr4 family |
| CAP2UW1_2202 | 2484845 | 2486026 | CRISPR-associated protein, Cmr3 family |
| CAP2UW1_2203 | 2486026 | 2488923 | CRISPR-associated protein, Crm2 family |
| CAP2UW1_2204 | 2488920 | 2490029 | hypothetical protein |
| CAP2UW1_2205 | 2490145 | 2491347 | CRISPR-associated protein, NE0113 family |
| CAP2UW1_2426 | 2788299 | 2789519 | major facilitator superfamily MFS_1 |
| CAP2UW1_2427 | 2789566 | 2790006 | thioesterase superfamily protein |
| CAP2UW1_2475 | 2844093 | 2844581 | ErfK/YbiS/YcfS/YnhG family protein |
| CAP2UW1_2476 | 2844578 | 2845039 | CMP/dCMP deaminase zinc-binding |
| CAP2UW1_2477 | 2845199 | 2845714 | cyclic nucleotide-binding protein |
| CAP2UW1_2514 | 2899117 | 2901846 | pyruvate phosphate dikinase |
| CAP2UW1_2644 | 3042914 | 3044902 | hypothetical protein |
| CAP2UW1_2788 | 3226790 | 3228124 | MATE efflux family protein |
| CAP2UW1_2932 | 3388367 | 3389566 | lipolytic protein G-D-S-L family |
| CAP2UW1_3041 | 3499373 | 3500983 | methyl-accepting chemotaxis sensory transducer |
| CAP2UW1_3078 | 3541631 | 3542821 | cation diffusion facilitator family transporter |
| CAP2UW1_3246 | 3737055 | 3738011 | hypothetical protein |
| CAP2UW1_3249 | 3741918 | 3743177 | O-antigen polymerase |
| CAP2UW1_3343 | 3841585 | 3843078 | acyltransferase, WS/DGAT/MGAT |
| CAP2UW1_3358 | 3857175 | 3858671 | AAA ATPase |
| CAP2UW1_3368 | 3868051 | 3868302 | prevent-host-death family protein |
| CAP2UW1_3369 | 3868299 | 3868553 | addiction module toxin, Txe/YoeB family |
| CAP2UW1_3379 | 3880066 | 3880839 | hypothetical protein |
| CAP2UW1_3543 | 4052499 | 4052918 | hypothetical protein |
| CAP2UW1_3544 | 4053064 | 4053258 | transcriptional regulator, ArsR family |
| CAP2UW1_3545 | 4053454 | 4053630 | hypothetical protein |
| CAP2UW1_3546 | 4053735 | 4054145 | glutathione-dependent formaldehyde-activating GFA |
| CAP2UW1_3547 | 4054250 | 4054681 | hypothetical protein |
| CAP2UW1_3548 | 4054769 | 4055440 | hypothetical protein |
| CAP2UW1_3549 | 4055532 | 4055690 | hypothetical protein |
| CAP2UW1_3563 | 4066489 | 4067730 | hypothetical protein |
| CAP2UW1_3564 | 4067860 | 4069695 | Carbamoyltransferase |
| CAP2UW1_3565 | 4069698 | 4069847 | hypothetical protein |
| CAP2UW1_3566 | 4069859 | 4070254 | hypothetical protein |
| CAP2UW1_3567 | 4070385 | 4070579 | hypothetical protein |
| CAP2UW1_3568 | 4070669 | 4071001 | RNP-1 like RNA-binding protein |
| CAP2UW1_3585 | 4092292 | 4092480 | Rubredoxin-type Fe(Cys)4 protein |
| CAP2UW1_3586 | 4092572 | 4092967 | response regulator receiver protein |
| CAP2UW1_3587 | 4093011 | 4093373 | response regulator receiver protein |
| CAP2UW1_3588 | 4093388 | 4093897 | CheW protein |
| CAP2UW1_3589 | 4093970 | 4096135 | methyl-accepting chemotaxis sensory transducer |
| CAP2UW1_3590 | 4096176 | 4101569 | CheA signal transduction histidine kinase |
| CAP2UW1_3615 | 4123864 | 4124661 | prolipoprotein diacylglyceryl transferase |
| CAP2UW1_3616 | 4124700 | 4126085 | Malonyl-CoA decarboxylase |
| CAP2UW1_3617 | 4126138 | 4126611 | putative lactoylglutathione lyase |
| CAP2UW1_3725 | 4264256 | 4264387 | hypothetical protein |
| CAP2UW1_3726 | 4264474 | 4264578 | hypothetical protein |
| CAP2UW1_3790 | 4334644 | 4335444 | hypothetical protein |
| CAP2UW1_3791 | 4335441 | 4336103 | Cobyrinic acid ac-diamide synthase |
| CAP2UW1_3880 | 4430120 | 4432720 | PII uridylyl-transferase |
| CAP2UW1_3881 | 4432717 | 4433145 | hypothetical protein |
| CAP2UW1_3946 | 4509135 | 4509368 | hypothetical protein |
| CAP2UW1_3947 | 4509365 | 4512133 | diguanylate cyclase/phosphodiesterase with PAS/PAC sensor(s) |
| CAP2UW1_3996 | 4553549 | 4554382 | hypothetical protein |
| CAP2UW1_3997 | 4554379 | 4555218 | putative signal peptide protein |
| CAP2UW1_3998 | 4555224 | 4556915 | putative signal peptide protein |
| CAP2UW1_3999 | 4556915 | 4561447 | alpha-2-macroglobulin domain protein |
| CAP2UW1_4000 | 4561492 | 4562208 | protein of unknown function DUF1175 |
| CAP2UW1_4001 | 4562208 | 4563896 | hypothetical protein |
| CAP2UW1_4140 | 4715767 | 4716696 | MJ0042 family finger-like protein |
| CAP2UW1_4141 | 4716702 | 4717592 | ribosomal protein L11 methyltransferase |
| CAP2UW1_4142 | 4717607 | 4718971 | acetyl-CoA carboxylase biotin carboxylase subunit |
| CAP2UW1_4143 | 4718987 | 4719442 | acetyl-CoA carboxylase, biotin carboxyl carrier protein |
| CAP2UW1_4144 | 4719519 | 4719701 | hypothetical protein |
| CAP2UW1_4145 | 4719753 | 4720304 | alkyl hydroperoxide reductase/ Thiol specific antioxidant/ Mal allergen |
| CAP2UW1_4146 | 4720315 | 4720908 | hypothetical protein |
| CAP2UW1_4179 | 4755383 | 4756975 | NAD(P) transhydrogenase subunit alpha |
| CAP2UW1_4180 | 4756988 | 4758406 | NAD(P)(+) transhydrogenase (AB-specific) |
| CAP2UW1_4181 | 4758629 | 4759090 | CBS domain containing protein |
| CAP2UW1_4182 | 4759148 | 4760269 | deoxyguanosinetriphosphate triphosphohydrolase |
| CAP2UW1_4183 | 4760262 | 4761350 | 3-dehydroquinate synthase |
| CAP2UW1_4184 | 4761338 | 4761874 | shikimate kinase |
| CAP2UW1_4229 | 4804587 | 4805612 | restriction endonuclease |
| CAP2UW1_4230 | 4805709 | 4809569 | type III restriction protein res subunit |
| CAP2UW1_4231 | 4809592 | 4810371 | putative restriction endonuclease |
| CAP2UW1_4250 | 4846380 | 4847870 | putative sigma54 specific transcriptional regulator |
| CAP2UW1_4251 | 4848033 | 4848422 | hypothetical protein |
| CAP2UW1_4252 | 4848419 | 4849819 | Radical SAM domain protein |
| CAP2UW1_4400 | 4990054 | 4990764 | hypothetical protein |
| CAP2UW1_4401 | 4990870 | 4991799 | hypothetical protein |
| CAP2UW1_4414 | 5002517 | 5002855 | hypothetical protein |
| CAP2UW1_4415 | 5003144 | 5003386 | protein of unknown function DUF433 |
| CAP2UW1_4416 | 5003383 | 5003739 | hypothetical protein |
| CAP2UW1_4417 | 5004291 | 5005292 | hypothetical protein |
| CAP2UW1_4418 | 5005507 | 5005701 | hypothetical protein |
| CAP2UW1_4419 | 5005915 | 5006286 | Antibiotic biosynthesis monooxygenase |
| CAP2UW1_4420 | 5006392 | 5006796 | Glyoxalase/bleomycin resistance protein/dioxygenase |
| CAP2UW1_4429 | 5013397 | 5013867 | hypothetical protein |
| CAP2UW1_4443 | 5028367 | 5028978 | cold-shock DNA-binding domain protein |
